# Inhibition of Non-small Cell Lung Cancer by Ferroptosis and Apoptosis Induction through P53 and GSK-3β/Nrf2 Signal Pathways using Qingrehuoxue Formula

**DOI:** 10.7150/jca.79465

**Published:** 2023-01-16

**Authors:** Fei Xu, Jingtao Zhang, Lingyun Ji, Wenqiang Cui, Jie Cui, Zhao Tang, Ning Sun, Guangming Zhang, Minghao Guo, Baojun Liu, Jingcheng Dong

**Affiliations:** 1 Department of Geriatric Medicine, Affiliated Hospital of Shandong University of Traditional Chinese Medicine 250014, Jinan, China.; 2 First Clinical Medical College, Shandong University of Traditional Chinese Medicine, Jinan 250014, China.; 3 College of Traditional Chinese Medicine, Shandong University of Traditional Chinese Medicine, Jinan 250355, China.; 4 Department of Neurology, Affiliated Hospital of Shandong University of Traditional Chinese Medicine, Jinan 250014, China.; 5 Department of Integrative Medicine, Huashan Hospital of Fudan University, Shanghai 200032, China.; 6 Department of Traditional Chinese Medicine, Nanjing Drum Tower Hospital, the Affiliated Hospital of Nanjing University Medical School, Nanjing 210008, China.

**Keywords:** Traditional Chinese Medicine, NSCLC, anti-tumor, mechanism, signal pathway

## Abstract

This study aimed to elucidate the effects of Qingrehuoxue Formula (QRHXF) on NSCLC and its underlying mechanisms. Nude mouse model of subcutaneous tumors was established. QRHXF and erastin were administered orally and intraperitoneally, respectively. Mice's body weight and subcutaneous tumor volumes were measured. The effects of QRHXF on epithelial-mesenchymal transition (EMT), tumor-associated angiogenesis and matrix metalloproteinases (MMPs) were assessed. Importantly, we also analysed the anti-NSCLC of QRHXF form the aspect of ferroptosis and apoptosis and investigate its underlying mechanisms. The safety of QRHXF in mice was also evaluated. QRHXF slowed down the speed of tumor growth and visibly inhibited tumor growth. The expression levels of CD31, VEGFA, MMP2 and MMP9 were prominently suppressed by QRHXF. Furthermore, QRHXF appeared to remarkably inhibite cell proliferation and EMT by decreasing Ki67, N-cadherin and vimentin expression but elevating E-cadherin expression. There were more apoptotic cells in QRHXF group's tumor tissues, and QRHXF treatment increased BAX and cleaved-caspased 3 levels but decreased Bcl-2 levels. QRHXF significantly increased the accumulation of ROS, Fe^2+^, H_2_O_2_, and MDA while reduced GSH levels. SLC7A11 and GPX4 protein levels were considerably suppressed by QRHXF treatment. Moreover, QRHXF triggered ultrastructural changes in the mitochondria of tumor cells. The levels of p53 and p-GSK-3β were upregulated, whereas that of Nrf2 was downregulated in the groups treated with QRHXF. QRHXF displayed no toxicity in mice. QRHXF activated ferroptosis and apoptosis to suppress NSCLC cell progression via p53 and GSK-3β/Nrf2 signaling pathways.

## Introduction

Lung cancer is a leading cause of death worldwide, with high morbidity and mortality rates. The number of lung cancer-related deaths was approximately 1.80 million in 2020 1. Accumulating evidence indicates that ferroptosis, a dynamic tumor suppressor, is closely involved in cancer occurrence and development. Ferroptosis is a novel type of programmed cell death, which differs from apoptosis, necrosis, and autophagy. It is based on iron-dependent lipid peroxidation and accumulation of reactive oxygen species (ROS) 2, which leads to mitochondrial damage and breakdown of membrane integrity. This subsequently facilitates cell disintegration and death. Ferroptosis is mainly regulated by glutathione peroxidase 4 (GPX4) 3, which is at the center of a network about regulatory mechanism of ferroptosis and plays a vital role in catalyzing the reduction of lipid peroxides. It is also accompanied by decreased solute carrier family 7 member 11 (SLC7A11) (the gene encoding for xCT) and glutathione (GSH) levels. Inducing ferroptosis provides new therapeutic opportunities for cancer management, particularly for conventional therapy-resistant cancers.

Numerous ferroptosis-associated pathways and transcription factors have been identified in the past few years, including tumor protein P53 (p53) and nuclear factor-E2-related factor 2 (Nrf2). Various studies have revealed that p53 is not only a tumor suppressor but also a ferroptosis regulator that enhances it by inhibiting SLC7A11 expression 4. Nrf2, a crucial anti-ferroptotic transcription mediator, translocates from the cytosol into the nucleus, binds to the antioxidant response element (ARE), and subsequently activates the transcription of downstream genes, including SLC7A11 and GPX4 5. Inactivation of glycogen synthase kinase-3β (GSK-3β) phosphorylation promotes nuclear accumulation of Nrf2^6^. Moreover, p53 and GSK3β/Nrf2 signaling pathways have also been implicated in apoptosis by promoting the release of pro-apoptotic factors 7, 8.

“Qing-re-huo-xue” formula (QRHXF), which includes *Scutellaria baicalensis* and *Radix paeoniae rubra*, is commonly used to treat chronic obstructive pulmonary disease (COPD) and lung cancer in traditional Chinese medicine. Our previous studies have demonstrated that QRHXF treatment markedly inhibited tumor growth and metastasis in a mouse lung cancer model 9, 10. QRHXF mainly includes eight ingredients: *albiflorin*, *paeoniflorin*, *baicalin*, *oroxylinA-7-O-glucuronide*, *wogonoside*, *baicalein*, *wogomn*, and *oroxylin A*. Most of these chemical components regulate the biological process of oxidative stress and affect antioxidant balance 11, 12. Hence, the present study investigated whether QRHXF inhibits tumor growth in lung cancer by inducing apoptosis and triggering ferroptosis in lung cancer cells using a murine lung cancer model.

## Materials and methods

### Mice

Male BALB/c nude mice (SPF, 5-week-olds) were purchased from Beijing Weitonglihua Laboratory Animal Co., Ltd. (Beijing, China). They were housed in a specific facility without pathogens, under a 12 h light-dark cycle, and food and water were provided ad libitum. All animals were allowed to acclimatize to their surroundings for 1 week. This study was approved by the Animal Care and Use Committee of the Affiliated Hospital of Shandong University of Traditional Chinese Medicine (2021-50).

### Establishment of model

A549 cells in the logarithmic growth phase were suspended in hosphate Buffer Saline (PBS) mixed with Matrigel, and the concentration was adjusted to 6×10^7^/ml. BALB/c nude mice were injected subcutaneously with 6×10^6^ A549 cells into the right axillary region. On day 15, the tumor masses were palpable, and the tumor volume reached approximately 200 mm^2^. The tumor-bearing mice were then blindly randomized into four groups: (1) control (Control), (2) erastin (ferroptosis activator) treatment (Erastin), (3) low-dose QRHXF (Low), and (4) high-dose QRHXF (High) groups. Body weight and subcutaneous tumor volume were measured once every 3 days. The tumor volume was calculated using the formula: volume (V)=a×b^2^/2 (where, a=maximum long diameter and b=maximum short diameter). After 21 days of intervention, the mice were sacrificed. Tumors and heart, liver, spleen, lung, and kidney tissues were extracted, and blood samples were collected. Additionally, tumor weights were measured, and images of the tumors were taken.

### QRHXF preparation and treatment

The Chinese herbs *Scutellaria baicalensis* and *Radix paeoniae rubra* were purchased from the Affiliated Hospital of Shandong University of Traditional Chinese Medicine. Pieces of these two herbs were mixed in a 3:2 (w/w) ratio and decocted as described in our previous study 9. The final dose of raw herbs for gavage was equivalent to 15 g/kg (herbs/body weight, high-dose group).

Mice in the Low and High groups were orally administered QRHXF once daily, while mice in the control and erastin groups received the same volume of sterilized water. Mice in the erastin group were injected intraperitoneally with 20 mg/kg of erastin every 3 days. Simultaneously, mice in the control, low, and high groups were administered the same amount of normal saline via intraperitoneal injection.

### Iron assay

Iron levels were determined using an iron assay kit (Nanjing Jiancheng, A039-2-1) according to the manufacturer's instructions. The tumor tissue (30 mg) was homogenized in normal saline and then centrifuged at 2500 rpm for 10 min at 4 °C to obtain the supernatant. Then, the supernatant and test solution were added to a tube and reacted with an iron chromogenic reagent at 100 °C for 5 min. After cooling to room temperature, the mixture was centrifuged at 3500 rpm for 10 min at 4 °C, and the supernatant was collected. Finally, 1.0 ml of the supernatant was taken to measure the absorbance values at 520 nm.

### Measurement of cellular ROS

ROS levels were measured using a tissue ROS assay kit (Baiaolaibo, HR8821). Fresh tumor tissues (50 mg) were added to a lysis buffer, and the supernatant was then obtained after being centrifuged at 100 g for 4 min at 4 °C. On the one hand, the protein concentration was measured using a BCA protein assay kit (Beyotime, P0010S); on the other hand, 200 uL of the sample was incubated with DHE for 30 min at 37 °C. Subsequently, fluorescence intensity was detected at an excitation wavelength of 488 nm and an emission wavelength of 610 nm. The results were expressed as the ratio of fluorescence intensity to protein concentration.

### Measurement of intracellular hydrogen peroxide (H_2_O_2_)

H_2_O_2_ levels were measured using an H_2_O_2_ assay kit (A064-1-1, Nanjing Jiancheng), according to the manufacturer's instructions. The tumor tissue (30 mg) was homogenized in normal saline and then centrifuged at 10000 rpm for 10 min at 4 °C to obtain the supernatant. The supernatant and test solution were then added to a tube and mixed with the H_2_O_2_ reaction liquid. The absorbance was measured at 405 nm.

### Measurement of GSH

The total GSH levels were quantified using a GSH assay kit (Beyotime, S0053), as described in the manufacturer's protocol. The tumor tissue (30 mg) was frozen with liquid nitrogen and ground into powder. A protein removing buffer M was added to the samples, and the suspension was centrifuged at 10000 g for 10 min at 4 °C to obtain the supernatant, which was prepared for the GSH assay. The samples were mixed with the detection working solution, and absorbance was measured at 412 nm. The total GSH content was calculated as the ratio of the absorbance value to the control group.

### Lipid peroxidation assessment

Malondialdehyde (MDA) assay kits (Beyotime, S0131M) were used to assess the level of lipid peroxidation according to the manufacturer's guidelines. Proteins were obtained from 30 mg of tumor tissues using a radioimmunoprecipitation assay (RIPA) protein extraction reagent (Beyotime, P0013B). The protein concentration was determined using a BCA protein assay kit. Then, 100 mL of the protein sample or standard protein was incubated with 200 mL of working fluid for MDA detection for 15 min at 100 °C. The samples were then cooled to room temperature and centrifuged at 1000 g for 10 min at room temperature to obtain the supernatant. Finally, 200 µL of the supernatant was added to the 96-well plate, and the absorbance was measured at 532 nm with a microplate reader. The MDA level was represented as the ratio of absorbance value to the control group.

### Transmission electron microscopy (TEM)

The tumors were retracted and immediately placed in a fixative (2.5% glutaraldehyde solution). Subsequently, three 1×1×1 mm slices were cut from different segments and post-fixed in the fixative. The samples were further processed by staining, dehydration, embedding, and sectioning to acquire ultrathin slices. Finally, the stained sections were visualized, and images were obtained using TEM (Hitachi Limited, Japan).

### Terminal deoxynucleotidyl transferase dUTP nick end labeling (TUNEL) assay

The tumor tissues were fixed with 4% paraformaldehyde and embedded in paraffin. Then, sections cut from tumor tissues were stained with TUNEL reagent (Beyotime, China, C1098) according to the manufacturer's protocol. The slides were viewed under a light microscope, and images from randomly selected areas were analyzed.

### Western blotting (WB)

Total protein was extracted from the tumor tissue using RIPA protein extraction reagent containing phosphatase and protease inhibitors. The total protein concentration was measured using a BCA Protein Assay Kit. Then, the protein samples were mixed with a loading buffer (containing β-mercaptoethanol) and were heated at 100 ℃ for 10 min. Immunoblotting was performed using Bcl2-Associated X Protein (BAX) (Abcam, ab32503), B-cell lymphoma 2 (Bcl-2) (Abcam, 182858), SLC7A11 (Huabio, HA600097), GPX-4 (Bioss, 3884R), p53 (Proteintech, 60283-2-1g), GSK-3β (Huabio, ET1607-71), p-GSK-3β (Bioss, 2066R), Nrf-2 (Santa Cruz, 365949), cluster of differentiation 31 (CD31) (Huabio, ER31219), vascular endothelial growth factor-A (VEGFA) (Huabio, ET1604-28), MMP2 (Proteintech, 10373-2-AP), MMP9 (Proteintech, 10375-2-AP), Histone-H3 (Proteintech, 17168-1-AP), and β-actin (Proteintech, HRP-60008). Densitometry was performed using ImageJ software.

### Immuohistochemical staining (IHC)

Tumor tissues were fixed in 4% paraformaldehyde and then embedded in paraffin. Sections cut from tissues were were dewaxed with xylene, rehydrated in ethanol and then stained with the E-cadherin (Huabio, EM0502), N-cadherin (Huabio, ET1607-37) and Vimentin (Huabio, ET-1610-39) antibodies overnight at 4 °C, respectively. The next day, the sections were stained with HRP-conjugated secondary antibodies and viewed under a fluorescence microscope. Representative images were captured and analyzed.

### Hematoxylin and eosin (HE) staining

The tumor, heart, liver, spleen, lung, and kidney tissues were fixed in 4% paraformaldehyde and embedded in paraffin. Then, 5 mm thick sections cut from the tissues were prepared and stained with HE, as described in the manuscript protocol. Tissue sections were observed, and images were captured under a light microscope.

### Statistical Analysis

SPSS v.12 software (IBM, Chicago, Illinois, USA) was used for the statistical analysis. Data are presented as mean ± standard deviation (SEM). Differences among groups were evaluated using a one-way analysis of variance (ANOVA). *P*-values <0.05 were considered statistically significant, and all experiments were performed at least thrice.

## Results

### QRHXF suppressed NSCLC tumorigenesis *in vivo*

**Figure 1:**
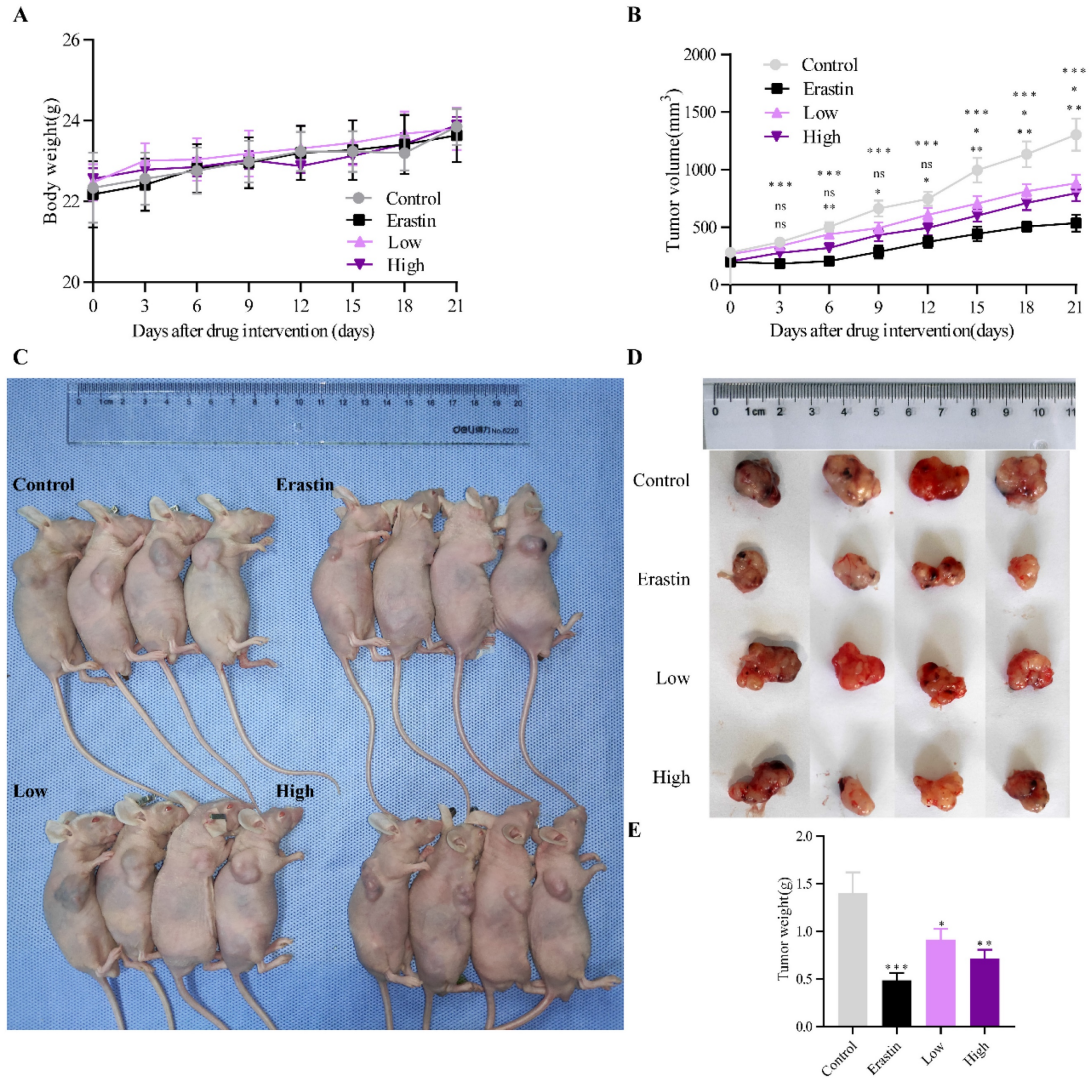
** QRHXF inhibited lung tumor growth in a subcutaneous lung cancer model. (A)** QRHXF had no effects on the weight of mice. **(B)**The growth curve of the tumor volume (mm^3^) in mice. QRHXF slowed down the speed of tumor growth. **(C and D)** The representative images of mice with subcutaneous tumors and xenograft tumors; The subcutaneous tumors in the nude mice treated with QRHXF (particularly high-dose) were noticeably smaller than those in the control mice. **(E)** The final weight of subcutaneous tumors. QRHXF significantly inhibited the growth of the subcutaneous tumors in nude mice. Data are presented as mean ± SEM (n=9 per group). Compared to the control group, ns, *P*>0.05, ^*^P<0.05, ^**^P<0.01, ^***^P<0.001.

A subcutaneous tumor model of NSCLC was established in male BALB/c mice to examine the antitumor effect of QRHXF. **Figure 1A** showed that QRHXF administration did not impact the mice's body weight (all* p*>0.05), indicating that QRHXF was not toxic to the mice. However, erastin and QRHXF significantly inhibited NSCLC cell growth (**Figure 1B-E**). Tumor volumes in the erastin and QRHXF groups were clearly suppressed compared to those in the control group (*p*<0.01, *p*<0.05; **Figure 1B-C**). Meanwhile, the weight of the tumors in the erastin- and QRHXF-treated groups was significantly lower than that in the control group (*p*<0.01, *p*<0.05; **Figure 1D-E**). With the growing doses of QRHXF as shown in **Figure 1B-E**, QRHXF groups showed a dose-dependent decline in tumor volume and weight. Moreover, it was worth mentioning that the antitumor activity of erastin was stronger than that of QRHXF. Considering these results together suggested that QRHFX inhibited NSCLC cell growth *in vivo.*

### QRHXF induced histopathological changes in tumor tissues

**Figure 2:**
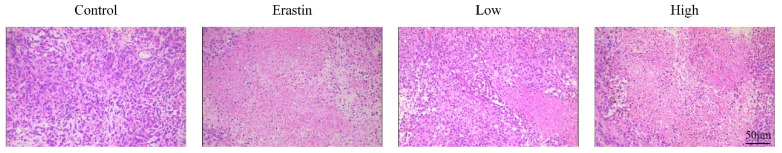
QRHXF induced histopathological changes in tumor tissues. Representative histological features of tumors examined by HE staining.

In the control group, HE staining of the tumor tissues showed that the tumor cells were tightly packed and exhibited a few necrotic cells. HE staining of the tumor tissues in the control group also showed obvious cell atypia, such as different cell sizes and diverse morphologies, increased nuclear size with dark staining, and irregular nuclear membranes (**Figure 2**). These results also indicated that the nude mouse models of subcutaneous tumors were successfully established. Cell density was markedly decreased in the erastin- and high-dose QRHXF-treated groups, and pathological karyokinesis was observed (**Figure 2**). Moreover, more cells showed a smaller nucleus with a shallower staining color, and there was more tumor necrosis in the tumor tissues (**Figure 2**). These observations further confirmed that QRHXF has antitumor activity.

### QRHXF inhibited tumor associated-angiogenesis and metastasis in tumor-bearing mice

**Figure 3:**
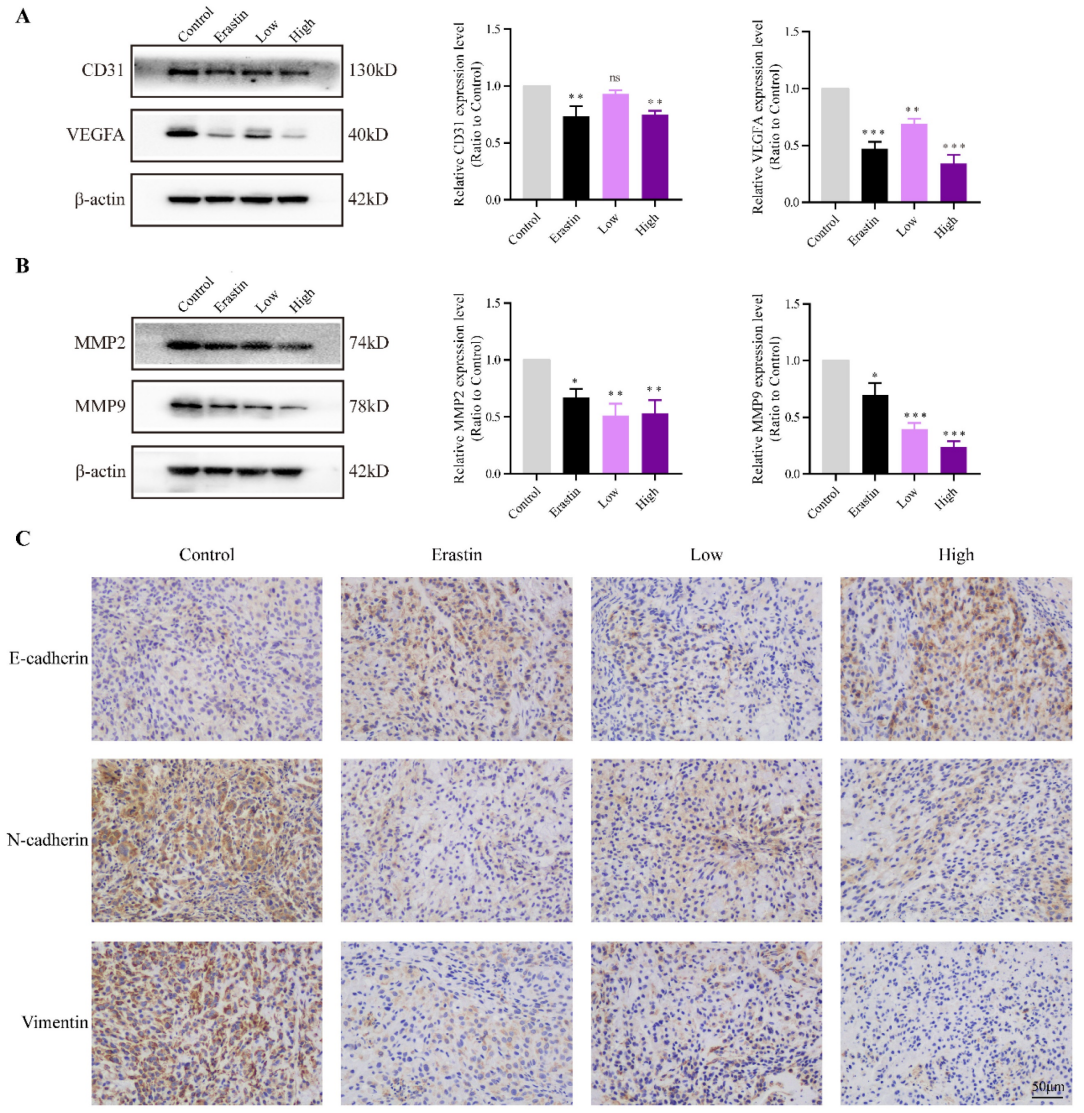
** QRHXF inhibited tumor associated-angiogenesis and metastasis in tumor-bearing nude mice. (A)** Western blotting showed that QRHXF down-regulated CD31 and VEGFA expression. **(B)** The protein expression levels of MMP2 and MMP9 measured by western blotting were significantly reduced by QRHXF treatment. **(C)** The results of immunohistochemical staining showed that QRHXF inhibited EMT as evidenced by elevating E-cadherin but decreasing N-cadherin and vimentin expression. Data are presented as mean ± SEM (n=4-5 per group). Compared to the control group, ns, *P*>0.05, ^*^P<0.05, ^**^P<0.01, ^***^P<0.001.

CD31 is an endothelial marker, and VEGFA is a key factor that induces tumor angiogenesis. The protein expression of CD31 and VEGFA in the Erastin and High-dose QRHXF groups was much lower than that in the control group (all *p*<0.01, **Figure 3A**).

EMT is a key mechanism related to the invasion, metastasis, and progression of NSCLC. The changes of E-cadherin, N-cadherin, vimentin and are matrix metallopeptidases are hallmarks of EMT. The results showed that the expression of MMP2 and MMP9 significantly decreased after treatment with erastin and QRHXF (***p*<0.05, *p*<0.01; Figure 3B**). Mice challenged with erastin exhibited less of the area of N-cadherin and vimentin stained cells bur more of the area of E-cadherin stained cells (**Figure 3C**). Similarly, high-dose QRHXF group also exhibited a significant increase of the area of positive E-cadherin staining while a remarkable reduction of the area of positive N-cadherin and vimentin staining, similar to erastin treated group (**Figure 3C**). Taken together, these findings suggested QRHXF had a strong weakening effect on tumor angiogenesis and metastasis of NSCLC cells.

### QRHXF induced apoptosis and inhibit proliferation of NSCLC cells in tumor-bearing mice

**Figure 4:**
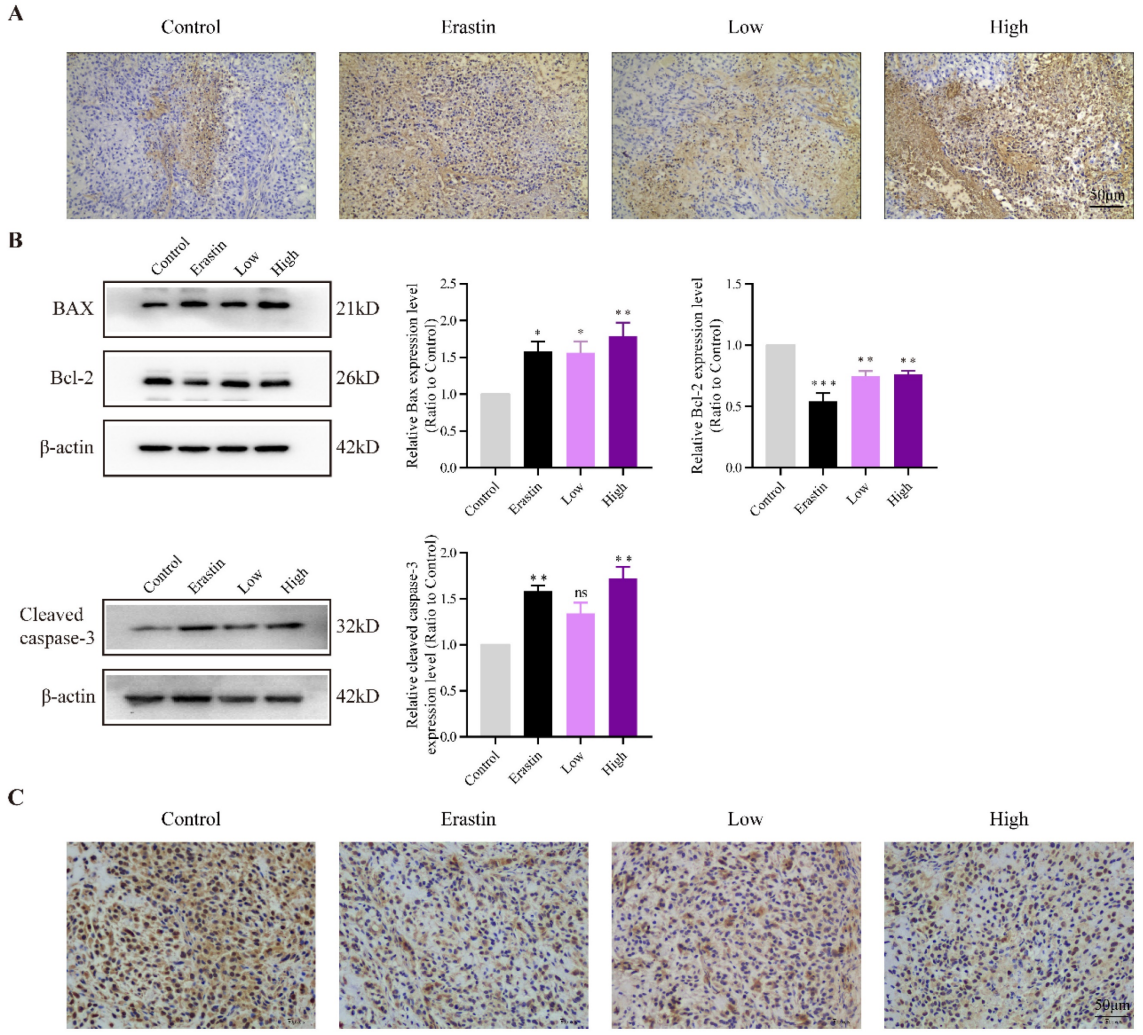
** QRHXF induced apoptosis and inhibited proliferation of NSCLC cells in tumor-bearing mice. (A)** TUNEL assay revealed that there were more apoptotic cells in tumor tissues of the QRHXF groups. **(B)** QRHXF treatment increased BAX and cleaved caspase-3 levels but decreased Bcl-2 levels. **(C)** The density of Ki67-positive cells in QRHXF-treated groups was significantly decreased compared with that in the control group. Data are presented as mean ± SEM (n=4 per group). Compared to the control group, ^*^P<0.05, ^**^P<0.01, ^***^P<0.001.

TUNEL staining showed more apoptotic cells in the erastin- and QRHXF-treated groups (**Figure 4A**), and apoptotic levels gradually increased with an increase in QRHXF concentration. The apoptosis-related factors, BAX and Bcl-2, were also markedly changed in the three treatment groups. **Figure 4B** showed that the protein levels of BAX increased in the Erastin and QRHXF groups (*p*<0.05, *p*<0.01), whereas Bcl-2 significantly decreased (*p*<0.01). Concurrently, with an increase in the expression of cleaved caspase-3 (**Figure 4B**), the effect of erastin and high-dose QRHXF activating apoptosis was confirmed. However, no differences were observed in the expression of cleaved caspase-3 between control group and low-dose QRHXF group.

Ki67 was used as an indicator related to the proliferation capacity of cells. The immunohistochemistry showed that the density of Ki67-positive cells in erastin and high-dose QRHXF group was signifcantly decreased compared with that in the control group (**Figure 4C**). Compared to low-dose QRHXF, high dose QRHXF exerted noticeable effects on inhibiting proliferation and promoting apoptosis of NSCLC cells. These results indicated that QRHXF contributed to the apoptosis and reduced cell proliferation of NSCLC cells *in vivo*.

### QRHXF promoted NSCLC cell ferroptosis in tumor-bearing mice

**Figure 5:**
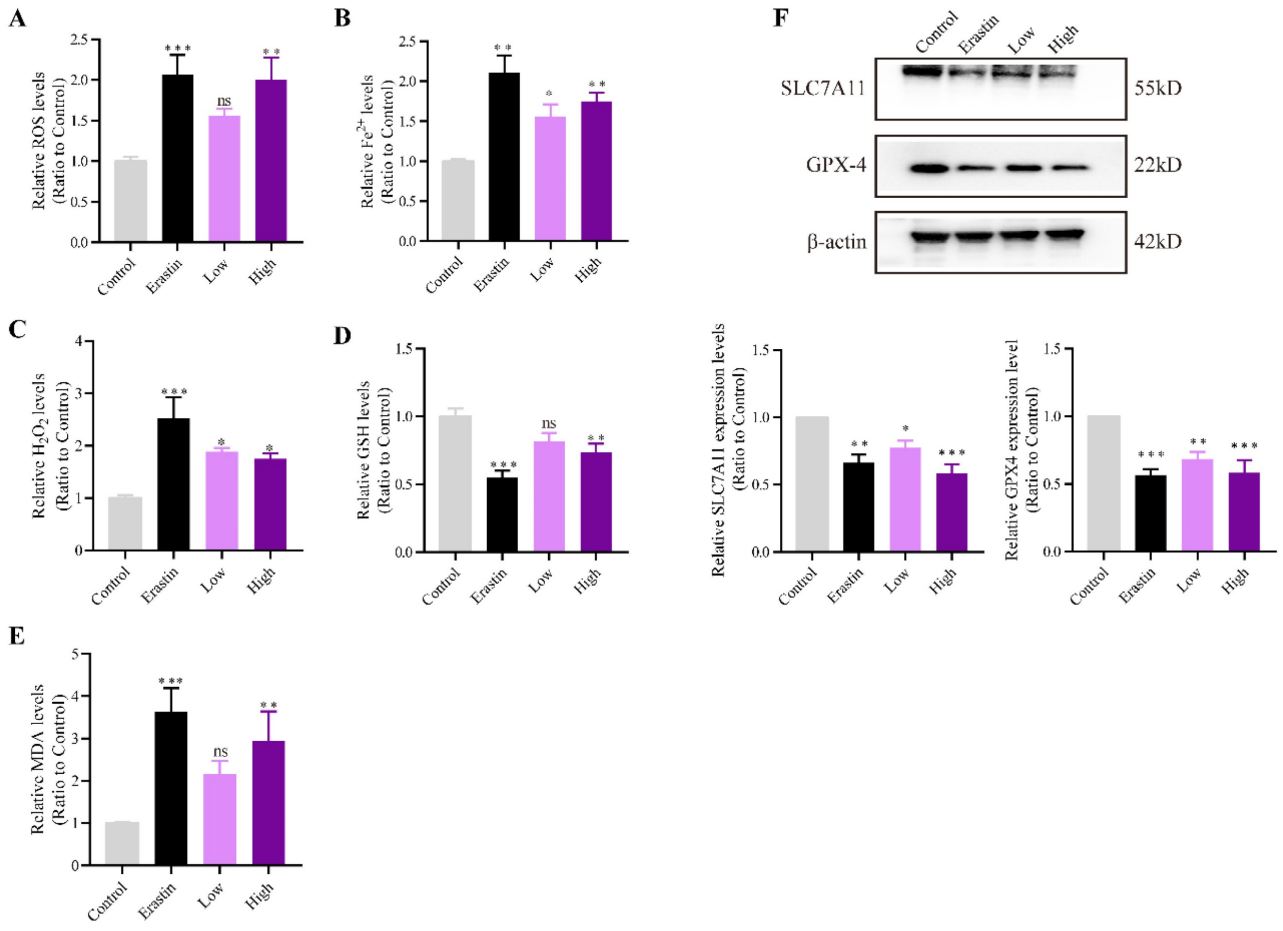
** QRHXF promoted NSCLC cell ferroptosis in tumor-bearing mice.** The ROS, iron, H_2_O^2^, GSH, and MDA levels were measured using assay kits. QRHXF significantly increased the accumulation of ROS **(A)**, iron **(B)**, H_2_O_2_ **(C)**, and MDA **(E)** but reduced GSH levels **(D)**. **(F)** Western blotting showed that SLC7A11 and GPX4 protein levels were considerably suppressed by QRHXF treatment. Data are presented as mean ± SEM (n=7-10 per group). Compared to the control group, ns, *P*>0.05, ^*^P<0.05,^ **^P<0.01, ^***^P<0.001.

ROS, H_2_O_2_, and the expression of other ferroptosis marker genes were measured to investigate whether QRHXF could promote ferroptosis of NSCLC cells *in vivo*. **Figure 5A** showed that intracellular ROS levels were increased after erastin and high-dose QRHXF treatment (*p*<0.01). However, no differences were observed in the level of ROS between control and low-dose QRHXF groups (p>0.05, **Figure 5A**). Iron and H_2_O_2_ were significantly upregulated by erastin and QRHXF (*p*<0.05, *p*<0.01; **Figure 5B and C**). In addition, erastin and high-dose QRHXF markedly decreased GSH levels but increased lipid peroxidation (MDA) levels (*p*<0.05, *p*<0.01; **Figure 5D and E**). Low-dose QRHXF didn't appeared to exert obvious effects on GSH or MDA levels in tumor tissues (both *p*>0.05,** Figure D and E**). Furthermore, erastin and QRHXF significantly inhibited the expression of SLC7A11 and GPX4 (*p*<0.05, *p*<0.01; **Figure 5F**). It was worth noting that erastin was more effective at inducing ferroptosis in NSCLC cells. These data suggested that QRHXF could trigger ferroptosis in NSCLC cells in tumor-bearing mice.

### QRHXF triggered ultrastructural changes of mitochondria in tumor tissues

**Figure 6:**
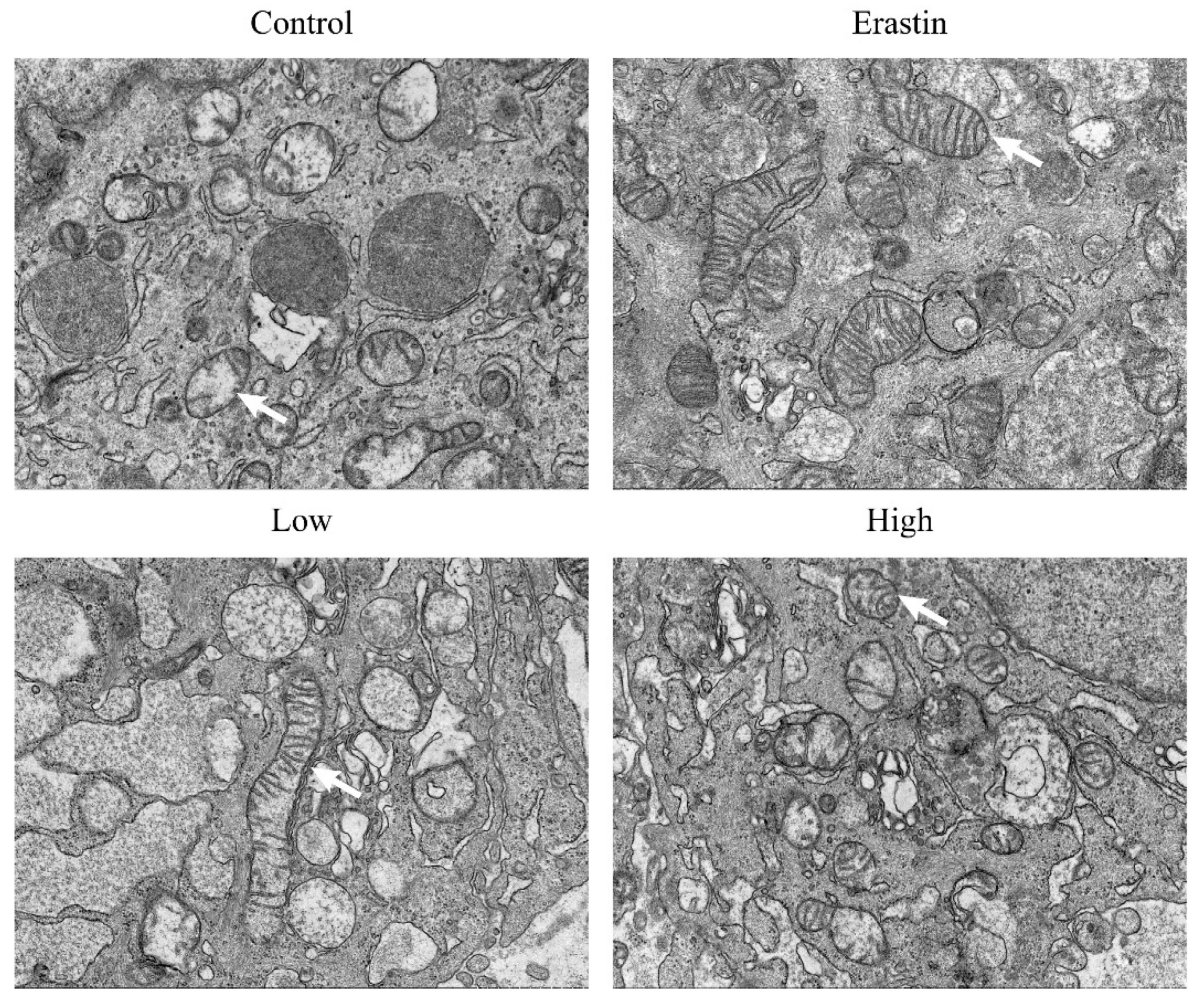
** QRHXF triggered ultrastructural changes of mitochondria in tumor tissues.** Representative transmission electron microscopy images showed that tumor tissues of mice in the QRHXF groups (especially those in the high-dose group) showed morphologic features of ferroptosis and apoptosis, shrunken mitochondria with increased membrane density, and decreased or disappeared mitochondrial cristae, and loss or disruption of cristae and swelling.

We performed TEM to observe the mitochondrial morphological changes in lung cancer cells. Strikingly, the tumor tissues of mice in the erastin and QRHXF groups showed morphologic features of ferroptosis, such as shrunken mitochondria with increased membrane density and decreased or disappeared mitochondrial cristae (**Figure 6**). However, erastin appeared to exert a more obvious morphological change during ferroptosis (**Figure 6**). Meanwhile, features of mitochondrial damage, such as loss or disruption of cristae and swelling, were also observed in the tumor tissues of mice treated with high-dose QRHXF (**Figure 6**).

### QRHXF induction of NSCLC cell apoptosis and ferroptosis through p53 and GSK-3β/Nrf2 signal pathways

**Figure 7:**
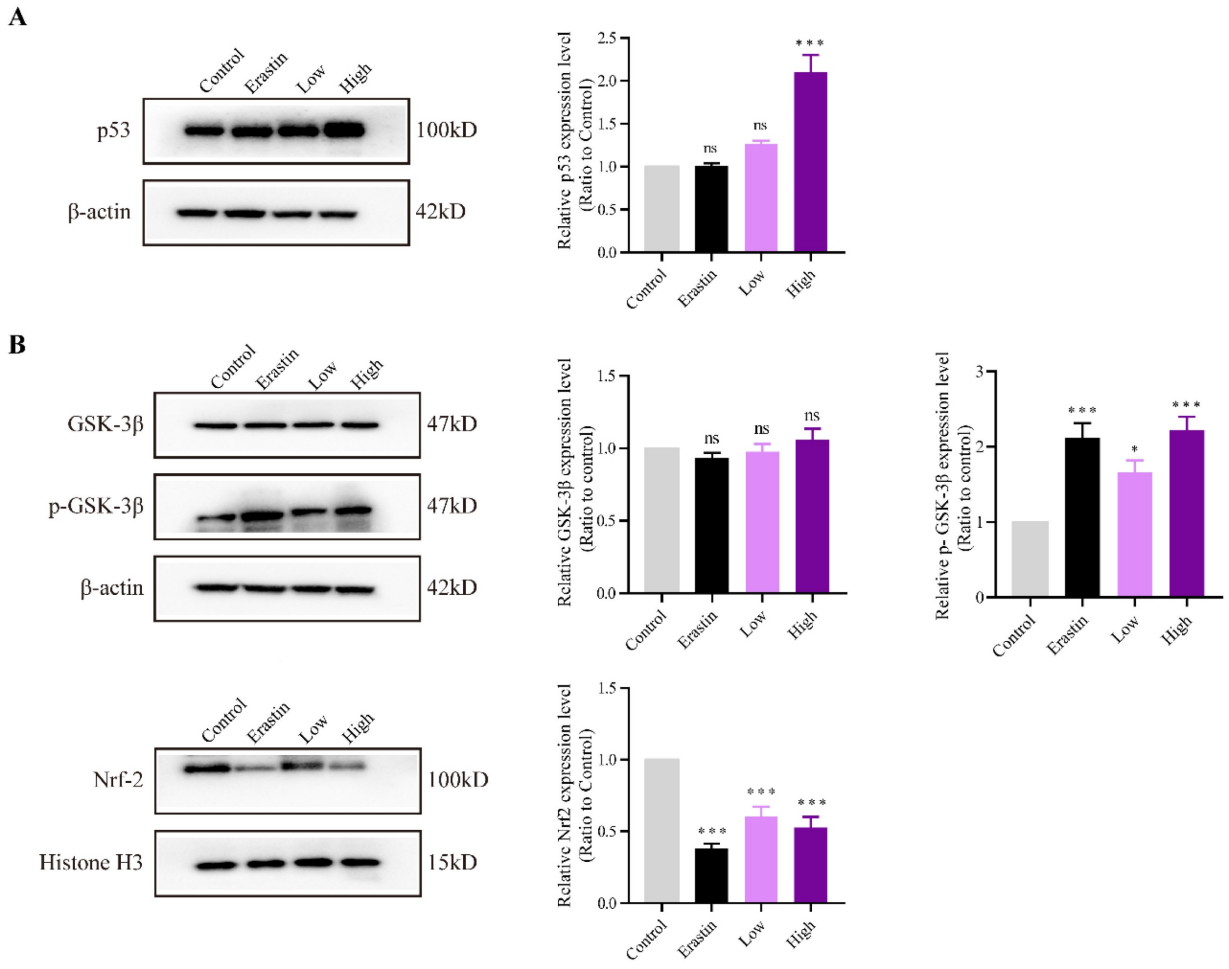
** QRHXF exerted effects on p53 and GSK-3β/Nrf2 signal pathways.** The protein expression levels of p53, GSK-3β, p-GSK-3β, and Nrf2 were measured by western blotting. QRHXF upregulated the protein levels of p53 **(A)** and p-GSK-3β **(B)** but downregulated nuclear Nrf2 in tumor tissues (B). No significant differences in GSK-3β levels were detected. Data are presented as mean ± SEM (n=4-5 per group). Compared to the control group, ns, *P*>0.05, ^*^P<0.05, ^***^P<0.001.

The expression levels of p53 and GSK-3β and the phosphorylation status of GSK-3β and Nrf2 were evaluated to explore the mechanisms underlying the effects of QRHXF on NSCLC cell apoptosis and ferroptosis. The results demonstrated that p53 expression was significantly increased in the high-dose QRHXF group compared with that in the control group (*p*<0.01; **Figure 7A**). In contrast, erastin and a low dose of QRXHF had no obvious effect on p53 expression (*p*>0.05; **Figure 7A**). Additionally, GSK-3β phosphorylation was significantly activated after treatment with erastin and QRHXF (*p*<0.01; **Figure 7B**). Furthermore, Nrf2 protein expression levels in the erastin and low- and high-dose QRHXF-treated groups were robustly decreased compared to those in the control group (*p*<0.01; **Figure 7B**). These observations indicated that QRHXF induced apoptosis and ferroptosis partly via p53 and GSK-3β/Nrf2 signal pathways.

### QRHXF displayed no toxicity in tumor-bearing mice

**Figure 8:**
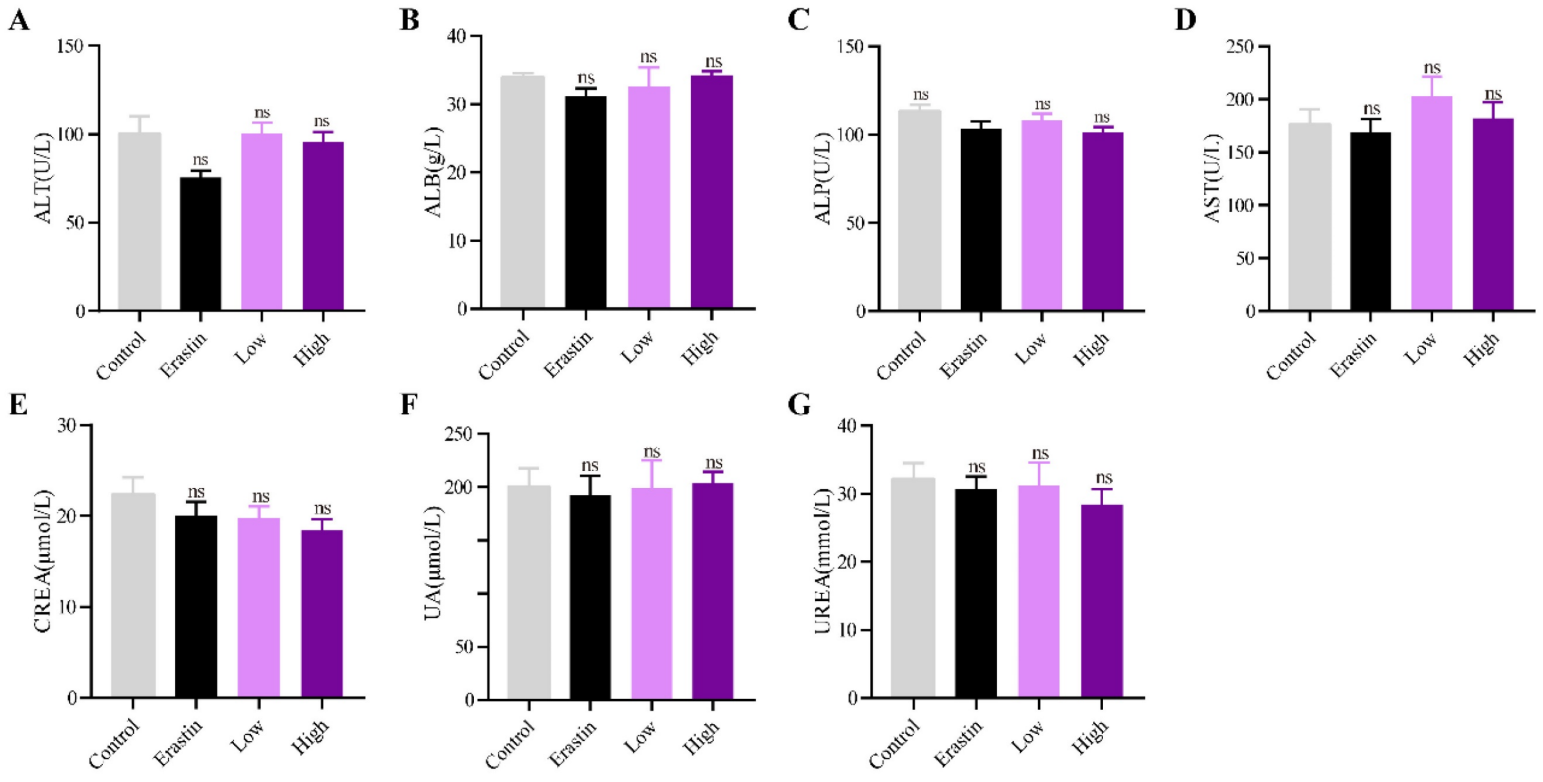
** QRHXF displayed no toxicity on the major organs in tumor-bearing mice.** The HE staining images of organs, including the lung, heart, liver, spleen, and kidneys, were analyzed, and no obvious pathologic changes were observed in these organs.

**Figure 9:**
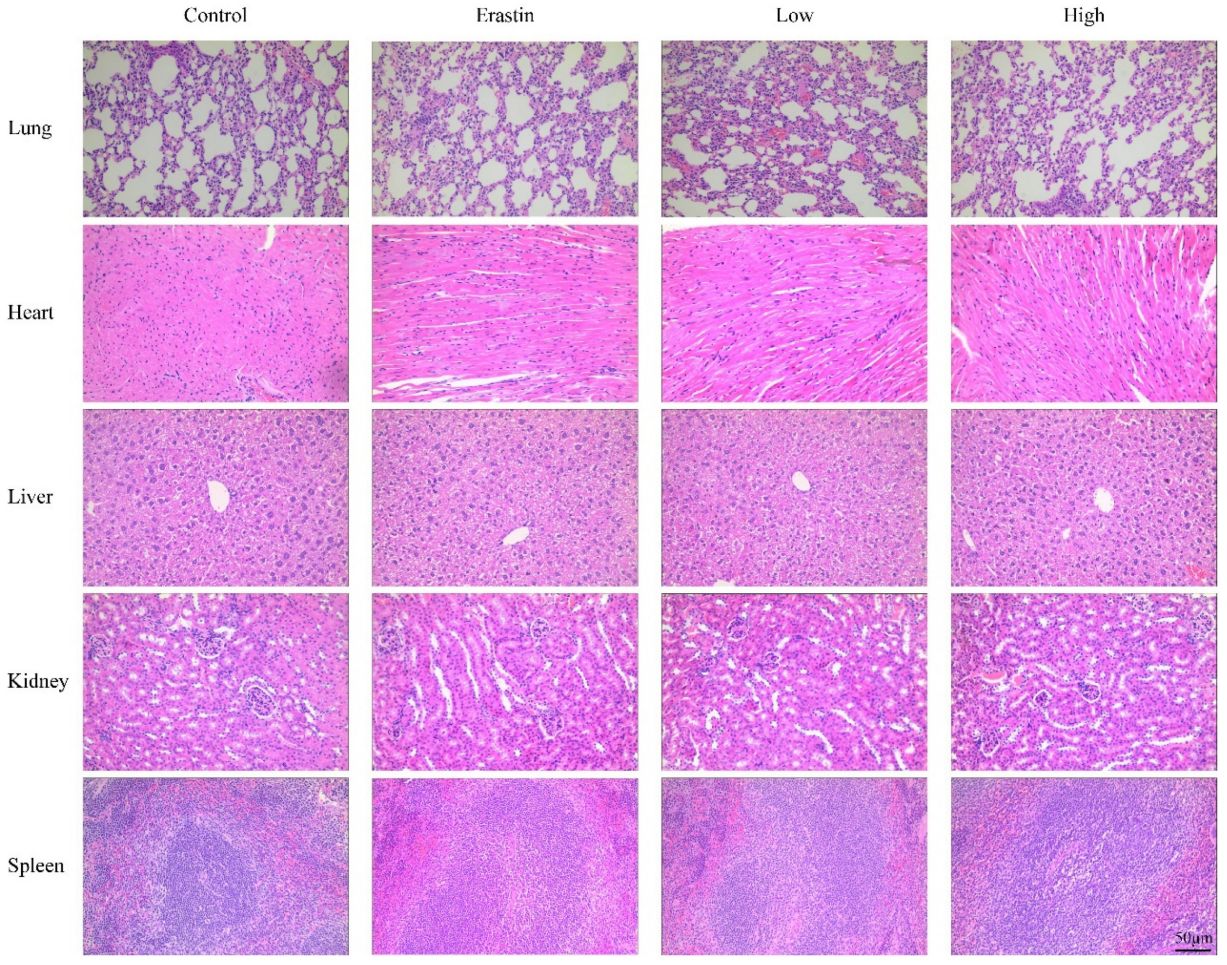
** QRHXF displayed no toxicity on liver and kidney functions.** Function indexes of the liver and kidney were tested, including ALT **(A)**, ALB **(B)**, ALP **(C)**, AST **(D)**, CREA **(E)**, UA **(F)**, and urea **(G)**. They were all found within the reference intervals, and no significant differences were observed among these groups. Data are presented as mean ± SEM (n=6-11 per group). Compared to the control group, ns, *P*>0.05.

The biological safety of QRHXF in mice was also investigated. HE staining showed no noticeable pathological changes in major organs, including the lung, heart, liver, spleen, and kidney (**Figure 8**). Liver and kidney function indices were also measured, including alanine aminotransferase (ALT), albumin (ALB), alkaline phosphatase (ALP), aspartate transaminase (AST), creatinine (CREA), UA, and urea. As shown in **Figure 9A-G,** QRHXF treatment did not affect liver or renal function (*p*>0.05). Additionally, no changes in body weight were observed in the two QRHXF groups compared to the control group (**Figure 1A**). Considering these results together clearly indicates that QRHXF caused no toxicity in the tumor-bearing mice.

## Discussion

QRHXF is frequently used in the clinical treatment of inflammatory diseases and cancer in Chinese. Our previous study demonstrated that QRHXF remarkably inhibit NSCLC cell growth and metastasis by ameliorating immunosuppressive tumor microenvironment 13. QRHXF as a developed herbal formula, contains a variety of chemical constituents, such as wogonin, baicalein, beta-sitosterol, baicalin, paeoniflorin, *et al.* 13. These ingredients exert antitumor effects by modulating oxidative stress and apoptosis and impeding cell cycle. These encouraging findings promoted us to valuate whether QRHXF could play antitumor effects by inducing ferroptosis. Data from this study revealed that QRHXF also suppressed NSCLC cell growth and metastasis by promoting apoptosis and ferroptosis through p53 and GSK-3β/Nrf2 signal pathways.

Apoptosis is considered programmed cell death, and most anticancer agents exert an inhibitory effect on cancers by reprogramming tumor cells to undergo apoptosis. In the present study, we evaluated whether QRHXF also could inhibit the growth of NSCLC cells by inducing apoptosis. Bcl-2, BAX and caspase 3 play crucial roles in apoptosis and their expression levels were evaluated by WB. TUNEL staining was performed to confirm this hypothesis. The results showed that QRHXF treatment induced more apoptotic cells and markedly upregulated BAX but downregulated Bcl-2. In addition, QRHXF upregulated the expression of cleaved caspase-3, thus inducing cellular apoptosis and promoting cell death. Baicalein, Scutellaria flavonoids, and other flavonoid components, as active ingredients of QRHXF, have been reported to have the potential to inhibit NSCLC cell proliferation and metastasis and activate autophagic flux 14-16. These effects may partly explain the anti-lung cancer efficacy of QRHXF in tumor-bearing mice.

MMPs, EMT and tumor angiogenesis are involved in tumor cell invasion and metastasis. As previously demonstrated, QRHXF effectively restrained NSCLC progression possibly by regulating RASAL2 gene 10. RASAL2 have been shown to be served as a regulator of EMT 17. Here, our results confirmed that QRHXF could suppress the process of EMT. Furthermore, QRHXF was able to remarkably inhibit the overproduction of CD31, VEGFA, MMP2 and MMP9. These data indicated that QRHXF exhibits extraordinary anti-metastasis effect in NSCLC.

Our previous studies demonstrated that baicalein could promote apoptosis in NSCLC cells via the ROS-mediated mitochondrial-endoplasmic reticulum pathway 18, 19. Excess production of mitochondrial ROS created by oxidative stress might lead to apoptosis via activation of caspases and damage to the mitochondrial membrane potential 20. In our study, ROS levels in tumor tissues were significantly elevated following QRHXF treatment in a subcutaneous lung cancer model. Hence, QRHXF may be a therapeutic approach for NSCLC by activating the apoptosis of cancer cells.

Compared to apoptosis, ferroptosis, which is a new form of cell death, has boosted the perspective of cancer strategies. Several studies implicate that oxidative stress, cysteine metabolism, and iron metabolism are involved in ferroptosis. Converging evidence indicates that excessive mitochondrial ROS accumulation leads to ferroptosis by promoting lipid peroxidation 21. QRHXF can ameliorate the oxidative-antioxidant balance, and our results showed that QRHXF treatment could elevate ROS and MDA levels in tumor tissues. In addition, excessive accumulation of intracellular Fe^2+^ further triggers the production of Fe^3+^ and ROS through Fenton reaction 21. Consistent with this, treatment with QRHXF was found to significantly elevate iron level. These observations prompted us to evaluate whether QRHXF modulated ferroptosis in lung cancer cells.

The SLC7A11-GPX4 signaling axis is considered the most dominant ferroptosis pathway. SLC7A11, the major component of system xc-, is mainly responsible for importing cystine, which is then reduced to cysteine and used to synthesize GSH 22. GPX4, the key upstream regulator in the ferroptotic process, exerts its function in suppressing lipid peroxidation by using GSH as a substrate 22. Inhibiting SLC7A11 leads to GSH depletion and blocks GPX4 activity 23, 24. Direct GPX4 inactivation or inhibition destroys the lipid peroxidation chain reaction 3, 23. Collectively inhibiting GSH synthesis or GPX4 activity contributes to ferroptotic cell death. Erastin is a typical inducer of ferroptosis, which triggers ferroptosis by inhibiting systemic xc-mediated cystine uptake. Consistent with previous studies 25, 26, our results suggested that erastin caused an increase in intracellular ROS but decreased GSH levels, accompanied by downregulation of SLC7A11 and GPX4. In this study, we also demonstrated that continued treatment with QRHXF could induce ferroptosis in NSCLC cells, characterized by elevated ROS, iron, H_2_O_2_, and MDA levels and lower intracellular GSH levels. Moreover, QRHXF promoted GPX4 degradation and inhibited SLC7A11 protein expression. Interestingly, electron microscopy revealed the morphological features of ferroptosis in the presence of QRHXF, suggesting that QRHXF exerted cytotoxic effects on NSCLC cells by targeting ferroptosis.

It is commonly accepted that p53 plays a central role in cell cycle arrest, apoptosis, and DNA damage. However, emerging research indicates that the tumor suppressor p53, a positive regulator of ferroptosis, promotes ferroptosis via a transcription- dependent mechanism 27-29. Previous experiments have shown that SLC7A11 is a direct p53 target, and p53 represses SLC7A11 expression, thereby mediating lipid peroxidation and ferroptotic cell death 29, 30. In addition, the previous study have shown that the activation of p53 depends on excessive ROS induced by erastin, which in turn gives a rise to ROS generation 31. In this context, erastin treatment behaved as expected in this respect, as we found an increase in ROS and p53 activation in tumor tissues in erastin-treated mice. Given that the administration of QRHXF significantly reduced SLC7A11 protein expression and led to ROS overproduction, here we investigated the relationship between QRHXF and p53. Similarly, we observed that QRHXF treatment markedly enhanced p53 expression and behaved the cytotoxic effects as expected in two respects, including the induction of apoptosis and ferroptosis in NSCLC cells.

Nrf2 is a transcription factor that plays a key role in regulating cellular oxidative steady-state by binding to the antioxidant response element (ARE) in the nucleus to promote target gene transcription. Emerging evidence has implicated that Nrf2 performed a protective role against ferroptosis 5, 32. Inhibition or knockdown of Nrf2 significantly reduces the expression of SLC7A11 and GPX4, resulting in an imbalance in homeostatic oxygen homeostasis 5, 32. Nrf2 can act via a direct or indirect effect on GPX4 expression and function. Previous studies confirmed that treatment with erastin significantly decreased the expression of Nrf2 by promoting Nrf2 degradation 33, 34. Consistent with the published studies 33, 34, we also found that erastin treatment remarkably reduced Nrf2, SCL7A11 and GPX4 protein expression. High-dose QRHXF approximately exterted similar effects of erastin, which might be due to the fact that A549 cells represent insensitive to ferroptosis induced by erastin^35^. Conversely, evidence also suggested that ferroptosis induced by erastin was reversed by Nrf2 inhibition through modulting heme oxygenase-1 (HO-1) 36, 37. However, HO-1 attenuated oxidative stress by decreasing overall ROS generation and promotes degradation of the pro-oxidant 38. It may be explained by the crosstalk between ROS and Nrf2 and Nrf2/HO-1 appears to play a dual role in redox homeostasis.

**Figure 10:**
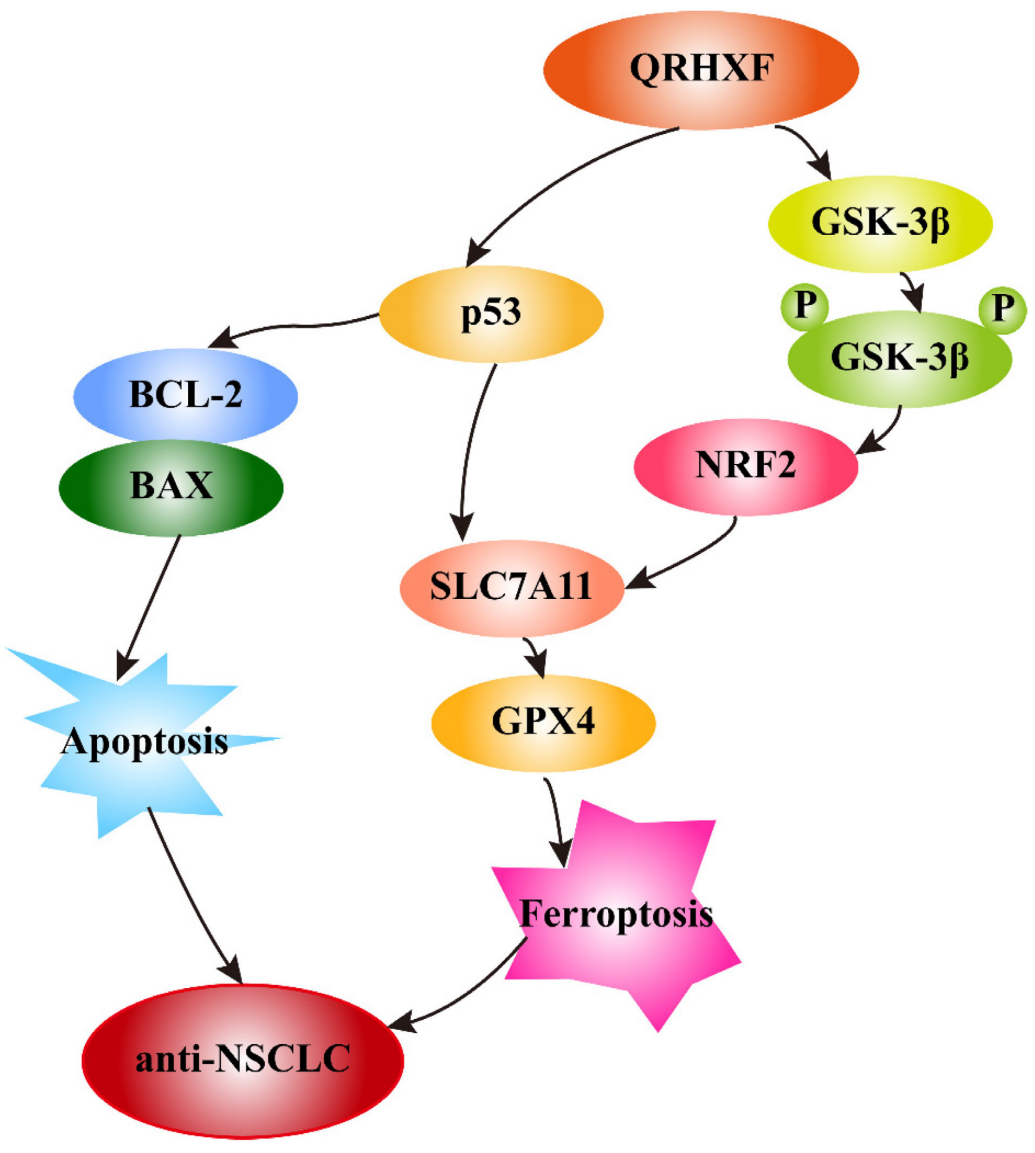
Schematic illustration of the proposed mechanisms by which QRHXF promoted ferroptosis and apoptosis in NSCLC cells.

In addition, growing evidence indicates that GSK-3β regulates the oxidant-antioxidant balance by modulating the transcription factor Nrf2 39, 40. Phosphorylated GSK3β activation is positively correlated with Nrf2 26, 41, and inhibition of GSK-3β phosphorylation abolishes Nrf2 activation 42. GSK-3β overexpression sensitizes erastin-induced ferroptosis, with an abundant accumulation of lipid peroxide and ROS production 26, 43. In turn, erastin-induced ferroptosis can be abrogated by GSK-3β, inhibition which is antagonized by inactivating Nrf2 26. Similarly, this study detected significant p-GSK3β elevation but decreased nuclear Nrf2 levels in tumor tissues treated with QRHXF. Thus, we inferred that the mechanism of ferroptosis promotion by QRHXF treatment might also be associated with the GSK-3β/Nrf2 signaling axis (**Figure 10**). However, the contribution of ferroptosis by QRHXF toward the observed anti-NSCLC activity is yet to be further explored.

## Conclusion

In this study, we demonstrated the potential therapeutic effect of QRHXF in mouse lung cancer model and explored its regulatory mechanism. This potential therapeutic effect might be mediated by inducing apoptosis and ferroptosis. At the same time, QRHXF also exerted anti-metastasis and anti-proliferation effects as evidenced by inhibiting EMT and tumor-associated angiogenesis and diminishing elevated expression of MMPs and Ki67. QRHXF activated apoptosis and ferroptosis in NSCLC cells partly via the p53 and GSK-3β/Nrf2 signaling pathways (**Figure 10**). Our study provided new insights into the application of QRHXF in lung cancer treatment.

## Abbreviations

HE: hematoxylin and eosin
CD31: cluster of differentiation 31
VEGFA: vascular endothelial growth factor-A
MMP2: matrix metallopeptidase-2
MMP9: matrix metallopeptidase
BAX: Bcl2-Associated X Protein
Bcl-2: B-cell lymphoma 2
TUNEL: Terminal deoxynucleotidyl transferase dUTP nick end labeling
ROS: reactive oxygen species
H_2_O_2_: hydrogen peroxide
GSH: glutathione
MDA: malondialdehyde
SLC7A11: solute carrier family 7 member 11
GPX4: glutathione peroxidase 4
GSK-3β: glycogen synthase kinase-3 beta
Nrf2: nuclear factor-E2-related factor 2
WB: western blotting

## References

[1] Sung H, Ferlay J, Siegel RL, Laversanne M, Soerjomataram I, Jemal A (2021). Global Cancer Statistics 2020: GLOBOCAN Estimates of Incidence and Mortality Worldwide for 36 Cancers in 185 Countries. CA Cancer J Clin.

[2] Stockwell BR, Friedmann AJ, Bayir H, Bush AI, Conrad M, Dixon SJ (2017). Ferroptosis: A Regulated Cell Death Nexus Linking Metabolism, Redox Biology, and Disease. Cell.

[3] Yang WS, SriRamaratnam R, Welsch ME, Shimada K, Skouta R, Viswanathan VS (2014). Regulation of ferroptotic cancer cell death by GPX4. Cell.

[4] Kuganesan N, Dlamini S, Tillekeratne L, Taylor WR (2021). Tumor suppressor p53 promotes ferroptosis in oxidative stress conditions independent of modulation of ferroptosis by p21, CDKs, RB, and E2F. J Biol Chem.

[5] Dodson M, Castro-Portuguez R, Zhang DD (2019). NRF2 plays a critical role in mitigating lipid peroxidation and ferroptosis. Redox Biol.

[6] Cuadrado A (2015). Structural and functional characterization of Nrf2 degradation by glycogen synthase kinase 3/β-TrCP. Free Radic Biol Med.

[7] Yan C, Zhang X, Miao J, Yuan H, Liu E, Liang T (2020). Farrerol Directly Targets GSK-3β to Activate Nrf2-ARE Pathway and Protect EA.hy926 Cells against Oxidative Stress-Induced Injuries. Oxid Med Cell Longev.

[8] Yu S, Guo L, Yan B, Yuan Q, Shan L, Zhou L (2022). Tanshinol suppresses osteosarcoma by specifically inducing apoptosis of U2-OS cells through p53-mediated mechanism. J Ethnopharmacol.

[9] Xu F, Cui W, Zhao Z, Gong W, Wei Y, Liu J (2017). Targeting Tumor Microenvironment: Effects of Chinese Herbal Formulae on Macrophage-Mediated Lung Cancer in Mice. Evid-Based Compl Alt.

[10] Lv Z, Chen X, Yang K, Zhao Y, Cui J, Tulake W (2022). Transcriptome Profiling of A549 Xenografts of Nonsmall-cell Lung Cancer Treated with Qing-Re-Huo-Xue Formula. Evid-Based Compl Alt.

[11] Lin MY, Cheng WT, Cheng HC, Chou WC, Chen HI, Ou HC (2021). Baicalin Enhances Chemosensitivity to Doxorubicin in Breast Cancer Cells via Upregulation of Oxidative Stress-Mediated Mitochondria-Dependent Apoptosis. Antioxidants (Basel).

[12] Han X, Hu S, Yang Q, Sang X, Tang D, Cao G (2022). Paeoniflorin ameliorates airway inflammation and immune response in ovalbumin induced asthmatic mice: From oxidative stress to autophagy. Phytomedicine.

[13] Xu F, Cui W, Zhao Z, Gong W, Wei Y, Liu J (2017). Targeting Tumor Microenvironment: Effects of Chinese Herbal Formulae on Macrophage-Mediated Lung Cancer in Mice. Evid Based Complement Alternat Med.

[14] Zhao Z, Liu B, Sun J, Lu L, Liu L, Qiu J (2019). Scutellaria Flavonoids Effectively Inhibit the Malignant Phenotypes of Non-small Cell Lung Cancer in an Id1-dependent Manner. Int J Biol Sci.

[15] Zhao Z, Liu B, Sun J, Lu L, Liu L, Qiu J (2019). Baicalein Inhibits Orthotopic Human Non-Small Cell Lung Cancer Xenografts via Src/Id1 Pathway. Evid Based Complement Alternat Med.

[16] Gong WY, Wu JF, Liu BJ, Zhang HY, Cao YX, Sun J (2014). Flavonoid components in Scutellaria baicalensis inhibit nicotine-induced proliferation, metastasis and lung cancer-associated inflammation *in vitro*. Int J Oncol.

[17] Li N, Li S (2014). RASAL2 promotes lung cancer metastasis through epithelial-mesenchymal transition. Biochem Biophys Res Commun.

[18] Deng X, Liu J, Liu L, Sun X, Huang J, Dong J (2020). Drp1-mediated mitochondrial fission contributes to baicalein-induced apoptosis and autophagy in lung cancer via activation of AMPK signaling pathway. Int J Biol Sci.

[19] Kiartivich S, Wei Y, Liu J, Soiampornkul R, Li M, Zhang H (2017). Regulation of cytotoxicity and apoptosis-associated pathways contributes to the enhancement of efficacy of cisplatin by baicalein adjuvant in human A549 lung cancer cells. Oncol Lett.

[20] Estaquier J, Vallette F, Vayssiere JL, Mignotte B (2012). The mitochondrial pathways of apoptosis. Adv Exp Med Biol.

[21] Zheng J, Conrad M (2020). The Metabolic Underpinnings of Ferroptosis. Cell Metab.

[22] Dixon SJ, Lemberg KM, Lamprecht MR, Skouta R, Zaitsev EM, Gleason CE (2012). Ferroptosis: an iron-dependent form of nonapoptotic cell death. Cell.

[23] Ursini F, Maiorino M (2020). Lipid peroxidation and ferroptosis: The role of GSH and GPx4. Free Radic Biol Med.

[24] Mandal PK, Seiler A, Perisic T, Kölle P, Banjac CA, Förster H (2010). System x(c)- and thioredoxin reductase 1 cooperatively rescue glutathione deficiency. J Biol Chem.

[25] Li Y, Zeng X, Lu D, Yin M, Shan M, Gao Y (2021). Erastin induces ferroptosis via ferroportin-mediated iron accumulation in endometriosis. Hum Reprod.

[26] Wu X, Liu C, Li Z, Gai C, Ding D, Chen W (2020). Regulation of GSK3β/Nrf2 signaling pathway modulated erastin-induced ferroptosis in breast cancer. Mol Cell Biochem.

[27] Chu B, Kon N, Chen D, Li T, Liu T, Jiang L (2019). ALOX12 is required for p53-mediated tumour suppression through a distinct ferroptosis pathway. Nat Cell Biol.

[28] Chen D, Tavana O, Chu B, Erber L, Chen Y, Baer R (2017). NRF2 Is a Major Target of ARF in p53-Independent Tumor Suppression. Mol Cell.

[29] Jiang L, Kon N, Li T, Wang SJ, Su T, Hibshoosh H (2015). Ferroptosis as a p53-mediated activity during tumour suppression. Nature.

[30] Kang R, Kroemer G, Tang D (2019). The tumor suppressor protein p53 and the ferroptosis network. Free Radic Biol Med.

[31] Zhao Y, Li Y, Zhang R, Wang F, Wang T, Jiao Y <p>The Role of Erastin in Ferroptosis and Its Prospects in Cancer Therapy</p>. 2020;Volume 13: 5429-41.

[32] Abdalkader M, Lampinen R, Kanninen KM, Malm TM, Liddell JR (2018). Targeting Nrf2 to Suppress Ferroptosis and Mitochondrial Dysfunction in Neurodegeneration. Front Neurosci.

[33] Shin D, Kim EH, Lee J, Roh JL (2018). Nrf2 inhibition reverses resistance to GPX4 inhibitor-induced ferroptosis in head and neck cancer. Free Radic Biol Med.

[34] Li Y, Yan H, Xu X, Liu H, Wu C, Zhao L (2020). Erastin/sorafenib induces cisplatin-resistant non-small cell lung cancer cell ferroptosis through inhibition of the Nrf2/xCT pathway. Oncol Lett.

[35] Gai C, Yu M, Li Z, Wang Y, Ding D, Zheng J (2020). Acetaminophen sensitizing erastin-induced ferroptosis via modulation of Nrf2/heme oxygenase-1 signaling pathway in non-small-cell lung cancer. J Cell Physiol.

[36] Kose T, Sharp PA, Latunde-Dada GO (2022). Upregulation of Nrf2 Signalling and the Inhibition of Erastin-Induced Ferroptosis by Ferulic Acid in MIN6 Cells. INT J MOL SCI.

[37] Gai C, Liu C, Wu X, Yu M, Zheng J, Zhang W (2020). MT1DP loaded by folate-modified liposomes sensitizes erastin-induced ferroptosis via regulating miR-365a-3p/NRF2 axis in non-small cell lung cancer cells. Cell Death Dis.

[38] Jaramillo MC, Zhang DD (2013). The emerging role of the Nrf2-Keap1 signaling pathway in cancer. Genes Dev.

[39] Yan C, Zhang X, Miao J, Yuan H, Liu E, Liang T (2020). Farrerol Directly Targets GSK-3β to Activate Nrf2-ARE Pathway and Protect EA.hy926 Cells against Oxidative Stress-Induced Injuries. Oxid Med Cell Longev.

[40] Zhang HF, Wang JH, Wang YL, Gao C, Gu YT, Huang J (2019). Salvianolic Acid A Protects the Kidney against Oxidative Stress by Activating the Akt/GSK-3β/Nrf2 Signaling Pathway and Inhibiting the NF-κB Signaling Pathway in 5/6 Nephrectomized Rats. Oxid Med Cell Longev.

[41] Lu H, Xiao H, Dai M, Xue Y, Zhao R (2022). Britanin relieves ferroptosis-mediated myocardial ischaemia/reperfusion damage by upregulating GPX4 through activation of AMPK/GSK3β/Nrf2 signalling. Pharm Biol.

[42] Li X, Zou Y, Xing J, Fu YY, Wang KY, Wan PZ (2020). Pretreatment with Roxadustat (FG-4592) Attenuates Folic Acid-Induced Kidney Injury through Antiferroptosis via Akt/GSK-3β/Nrf2 Pathway. Oxid Med Cell Longev.

[43] Wang L, Ouyang S, Li B, Wu H, Wang F (2021). GSK-3β manipulates ferroptosis sensitivity by dominating iron homeostasis. Cell Death Discov.

